# Effect of flaxseed oil supplementation on the erythrocyte membrane fatty acid composition and endocannabinoid system modulation in patients with coronary artery disease: a double-blind randomized controlled trial

**DOI:** 10.1186/s12263-020-00665-1

**Published:** 2020-05-05

**Authors:** Sevda Saleh-Ghadimi, Mohammad Alizadeh, Hamed Jafari-Vayghan, Masoud Darabi, Ali Golmohammadi, Sorayya Kheirouri

**Affiliations:** 1 Student Research Committee, Tabriz University of Medical Sciences, Tabriz, Iran; 2 Department of Clinical Nutrition, Faculty of Nutrition and Food Sciences, Tabriz University of Medical Sciences, Tabriz, Iran; 3 Faculty of Health, Arak University of Medical Sciences, Arak, Iran; 4 Department of Biochemistry and Clinical Laboratories, Faculty of Medicine, Tabriz University of Medical Sciences, Tabriz, Iran; 5 Cardiovascular Research Centre, Tabriz University of Medical Sciences, Tabriz, Iran; 6 Nutrition Research Center, Tabriz University of Medical Sciences, Tabriz, Iran

**Keywords:** CAD, Endocannabinoid, Flaxseed oil, CB1, CB2

## Abstract

**Background:**

The endocannabinoid system (ECS) overactivation, associated with increased inflammatory process, may act as a risk factor for coronary artery disease (CAD). Dietary fat may influence the ECS tone. The aim of the present study was to investigate the effect of flaxseed oil on the erythrocyte membrane fatty acid profile and ECS activity by the measurement of serum *N*-arachydonoil ethanolamine (AEA) and cannabinoid receptor type-1 (CB1), cannabinoid receptor type-2 (CB2), and fatty acid amide hydrolase (FAAH) mRNA expression.

**Methods:**

This clinical trial was performed on 44 patients with CAD. The intervention group received 1.5% fat milk supplemented with flaxseed oil (containing 2.5 g α-linolenic acid or ALA), while the placebo group received 1.5% fat milk for 10 weeks. The fatty acid profile of erythrocyte membrane phospholipids was measured by gas chromatography. The AEA level was determined using an ELISA kit, and real-time PCR was performed to measure CB1, CB2, and FAAH mRNA expression pre- and post-intervention.

**Results:**

Flaxseed oil supplementation resulted in a significant increase in the ALA content and a significant reduction in linoleic acid (LA) content of membrane phospholipids, compared to the placebo group (MD = − 0.35 and 2.89, respectively; *P* < 0.05). The within group analysis showed that flaxseed oil supplementation caused a significant reduction in both LA and arachidonic acid (MD = − 4.84 and − 4.03, respectively; *P* < 0.05) and an elevation in the ALA (MD = 0.37, *P* < 0.001) content of membrane phospholipids compared with the baseline. In the intervention group, a marked reduction was observed in the serum AEA level after 10 weeks of intervention, compared with the placebo group (MD = 0.64, *P* = 0.016). Changes in CB2 mRNA expression in the flaxseed oil group were significant (fold change = 1.30, *P* = 0.003), compared with the placebo group.

**Conclusion:**

Flaxseed oil supplementation could attenuate the ECS tone by decreasing the AEA level and increasing CB2 mRNA expression. Therefore, flaxseed oil may be considered a promising agent with cardioprotective properties.

## Introduction

Coronary artery disease (CAD) remains a major public health challenge [1]. There is now a growing body of evidence suggesting that lifestyle changes, with a focus on nutritional modifications, can help prevent or manage CAD [1–3]. Among numerous dietary approaches, the cardioprotective role of fat type (not its amount) has been highlighted [2]. Moreover, an overall risk reduction has been reported by replacing saturated fatty acids with polyunsaturated fatty acids (PUFAs) [4]. PUFAs consist of omega-3 and omega-6 fatty acids, the ratio of which is a risk indicator of CAD [5]. Fish oil supplementation, as a major source of omega-3 PUFAs, can reduce the omega-6/omega-3 ratio and exert cardioprotective effects [6, 7]. However, its consumption may be limited due to concerns about fish smell, taste, toxin content (e.g., methyl-mercury), eructation, allergies [1], and halitosis in high doses [8]. Therefore, incorporation of functional foods into diet seems to help promote compliance with a healthy dietary pattern and combat CAD.

Flaxseed oil, as the richest plant source of omega-3 (α-linolenic acid or ALA), can be a suitable alternative for balancing omega-6/omega-3 ratio [9]. Several findings suggest that flaxseed oil may act as a beneficial dietary supplement in the management of cardiometabolic disorders by exerting anti-inflammatory effects [10, 11] and improving the lipid profile [8, 12] and insulin resistance [11]. Enrichment of food with flaxseed oil has been suggested as a proper strategy for the dietary incorporation of flaxseed oil and fulfilling the omega-3 PUFA requirements [13]. Although the efficacy of ALA conversion is an issue, which should be considered in nutritional recommendations, several animal studies reported that eicosapentaenoic acid (EPA) and docosahexaenoic acid (DHA) elevated in a tissue-dependent manner [14]. Clinical trials have evaluated the potential elongation in the human blood, showing that high (> 5 g/day) [13, 15, 16] and low (< 5 g/day) [9] doses of dietary supplementation with flaxseed oil resulted in ALA and EPA elevation.

*N*-arachidonoyl-ethanolamine (AEA) and 2-arachidonoylglycerol (2-AG), known as endocannabinoids, are endogenously derived from arachidonic acid (AA) [17]. Therefore, incorporation of dietary oils with a high omega-3 content may help reduce AEA and 2-AG levels in the serum and tissues. AEA and 2-AG are ligands of the endocannabinoid system (ECS). Other components of this system include receptors, namely cannabinoid receptor type-1 (CB1) and cannabinoid receptor type-2 (CB2), and enzymes for biosynthesis and degradation of ligands [18]. Increased AEA and 2-AG levels may play a role in atherosclerosis development via platelet activation and prothrombotic effects [19]. Observational studies have revealed a correlation between elevated levels of AEA and 2-AG in hypercholesterolemic mice and proatherosclerotic effects [20]. Similar results have been reported in overweight and obese individuals [21, 22] with CAD, suggesting that endocannabinoids may serve as CAD risk factors.

CB1/CB2 receptors play different roles in atherosclerosis development. CB1 activation results in a proinflammatory response through production of reactive oxygen species in macrophages derived from human atheroma, whereas activation of CB2 receptors modulates immune challenges on immune cells [19] and decreases tumor necrosis factor-alpha (TNF-α) production which may suppress the atherosclerosis process [23, 24]. Therefore, the beneficial effects of omega-3-rich oils can be partly attributed to CB2-mediated effects in ECS.

In addition to the modulation of endocannabinoid tone, omega-3 fatty acids may act as precursors of bioactive endocannabinoid epoxides, such as eicosapentaenoyl ethanolamide (EPEA) and docosahexaenoyl ethanolamide (DHEA) products, which bind to CB1 and CB2 [18]. These two omega-3 long chain (LC)-PUFA-derived acyl conjugates (DHEA and EPEA) act as anti-inflammatory, vasodilatory, and anti-platelet aggregation agents, which may contribute to some of the positive effects of omega-3 PUFAs [25]. Moreover, in human inflammatory cells, DHEA appears to have greater affinity for CB2 than CB1, compared to AEA [26], indicating the enhanced anti-inflammatory effect of this compound. Therefore, it can be proposed that ECS acts as a missing link in promoting the beneficial effects of omega-3- rich oils in suppression of inflammation, atherosclerosis, and CAD [27].

Flaxseed oil supplementation increases ALA levels 2 weeks after the initiation of supplementation [1]. Subsequently, ALA by converting to EPA and DHA (to some extent) [28] can be a beneficial compound; this supports our hypothesis regarding the modulation of endocannabinoids tone. Therefore, in this study, we aimed to determine the effects of 10 weeks of intervention with 200 mL of milk, containing 5 g of flaxseed oil, on erythrocyte membrane lipids, and ECS activity by the measurement of serum AEA, CB1, CB2, and fatty acid amide hydrolase (FAAH) mRNA expression.

## Methods

### Study design

This two-arm parallel randomized controlled trial was conducted to evaluate the efficacy of a flaxseed oil-based intervention in CAD patients. All participants were recruited among individuals referred to Shahid Madani Hospital, affiliated to Tabriz University of Medical Sciences (TBZMED), Tabriz, Iran. The subjects were selected to participate in the study according to the following inclusion and exclusion criteria. The inclusion criteria were as follows: (1) voluntary participation in the study; (2) confirmed diagnosis of CAD by angiography, defined as the presence of ≥ 1 stenotic coronary artery with at least 50% stenosis; (3) being in the age range of 30–65 years; and (4) body mass index (BMI) of 25–35 kg/m^2^. On the other hand, the exclusion criteria were (1) development of myocardial infarction in the past six months; (2) diagnosis of uncontrolled diabetes, heart valve disease, or heart failure (function class III and IV); (3) regular use of immunosuppressive drugs, fish oil (omega-3), or fatty acid supplements; (4) consumption of weight loss drugs or history of weight loss surgery; (5) being pregnant or lactating; and (6) lactose deficiency (milk intolerance).

### Randomization and intervention

Eligible participants were randomly assigned to either the intervention or placebo group. The sequence of random allocation was generated, using a random sequence generator software. Each patient in the intervention group was compared with one control subject matched in terms of age, sex, BMI, and received medications (aspirin and statin). The participants were asked to consume either 200 mL of 1.5% fat milk/2.5% flaxseed oil emulsion (containing 2.5 g of ALA) per day as the intervention group or 1.5% fat milk per day as the placebo group for 10 consecutive weeks.

To prepare the milk/flaxseed oil emulsions, fresh flaxseed oil was extracted using the cold pressed method, and fatty acid profile of flaxseed oil was analyzed by gas chromatography (GC). The omega-3 and omega-6 compositions of flaxseed oil in this trial were as follows: linoleic acid (LA) 14.62%, ALA 50.86%, and omega-6/omega-3 ratio 0.28. In the next step, milk was used as the delivery system. Preparation of milk/flaxseed oil emulsion and packaging were performed by Pegah Dairy Co. (Tabriz, Iran), using high-pressure homogenization method. Each intervention pocket contained 200 mL of sterilized 1.5% fat milk + 2.5% flaxseed oil, and each placebo pocket contained 200 mL of sterilized 1.5% fat milk. Blinding was accomplished by labeling the pockets as “A” and “B” and addition of vanilla essence by the company.

The subjects were followed up via phone calls every week, and their compliance was checked. Milk pockets were given to the participants every 15 days. We asked them to record the number of unused milk pockets in a report form. To adjust the effect of diet on the study outcomes, all of the participants received a dietary plan with moderate calorie restriction during 10 weeks of intervention. The diet was composed of 55–60% carbohydrate, 30–35% lipid (with an emphasis on the type of fat), and 10–15% protein. A trained dietitian estimated the energy requirements and macronutrient distribution and trained the participants on the diet.

### Sample size

Based on the results of a study by Charles R. et al. [13], regarding the changes in EPA, we anticipated a between-group difference of 14.2. With an estimated dropout rate of 20%, the required sample size was measured to be 44 (22 patients per group), with an error of 5% and power of 90%.

### Measurements of anthropometric indices and blood pressure

Weight and height were measured to the nearest 0.1 kg, using a standardized digital column scale (Seca, Hamburg, Germany) and to the nearest 0.1 cm using a portable stadiometer (Seca, Hamburg, Germany), respectively. BMI was calculated by dividing weight in kilograms by height in meters squared. Waist circumference (WC) was also determined with a tape at the midpoint between the costal margin and the upper iliac crest, with subjects breathing normally. Moreover, neck circumference (NC) was measured below the cricoid cartilage. The physical activity level was measured using international physical activity questionnaire.

### Outcome measurements

At baseline and after 10 weeks of intervention, 5 mL blood samples were taken to evaluate the primary outcomes, including LC-fatty acids of membrane phospholipids, serum AEA level, and CB1, CB2, and FAAH mRNA expression after 10–12 hours of fasting. The level of LC-fatty acids was measured via GC to evaluate the effect of flaxseed oil on the omega-3/omega-6 ratio in the red blood cell membrane. Serum AEA levels were also measured by an ELISA kit (Bioassay Technology Laboratory, Shanghai Crystal Day Biotech Co., Ltd, Shanghai, China). Finally, real-time polymerase chain reaction (PCR) was used to evaluate CB1, CB2, and FAAH mRNA expression.

Whole blood samples were collected in non-EDTA-coated tubes. Sera were prepared from blood collection tubes after centrifugation at 500 g for 10 min and immediately frozen at − 80 °C.

### RNA isolation protocol for CB1, CB2, and FAAH genes

RNase-free protocols were followed throughout the study. Prior to total RNA extraction, the cells were lysed with lysis buffer. Next, RNAs were extracted using a NucleoSpin RNA kit (Macherey-Nagel, Düren, Germany) based on the manufacturer’s protocols. A NanoDrop spectrophotometer (NanoDrop One/Onec, Thermo Scientific) was employed for assessing RNA quality and quantity. Then, complementary DNA (cDNA) was produced from total RNA using the isolated total RNA, random hexamer primer, and reverse transcriptase, according to the manufacturer’s instructions (Thermo Scientific RevertAid First Strand cDNA Synthesis Kit, USA).

### Real-time PCR assay

SYBR Green Master Mix was used to examine the level of CB1, CB2, and FAAH mRNA expression. PrimerBank was also used for designing the primer sequences. The amount of mRNA expression was normalized against that of β-actin mRNA as the internal reference, and the relative expression of mRNA was calculated by determining the fold changes of parameters, computed as relative expression compared to the post-intervention stage in both intervention and placebo groups.

### Fatty acid extraction for GC analysis

Fatty acids were extracted from erythrocyte membrane phospholipids in three steps. The Bligh and Dyer’s method was used in the first step to extract total lipid from whole blood [29]. Next, an organic solvent, containing hexan/diethyl-ether/glacial acetic acid (70:30:1), and a silica gel plate were used to separate phospholipids via thin-layer chromatography. The phospholipids remained unchanged in this solvent system. In the third step, phospholipid fractions were extracted with a chloroform:methanol solution after scraping into glass tubes. Next, a direct transesterification method was applied to extract fatty acids from phospholipids. They were then analyzed by GC using a gas chromatograph (model 610, Buck Scientific) [30]. The isolated phospholipid fraction formed fatty acid methyl ester derivatives, which were separated on a TR-CN100 capillary column (60 × 0.25 mm). Tridecanoic acid (13:0) was used as the internal standard.

### Statistical analysis

Data were analyzed using SPSS, version 21. Normal distribution of data was examined based on Kolmogorov–Smirnov test. Data were expressed as mean (SD). Independent samples *t*-test was applied to evaluate between-group differences at baseline. Paired *t*-test was also used for comparing the baseline and post-intervention results within the groups. Moreover, analysis of covariance (ANCOVA) test was used to assess the mean differences between the groups after adjusting for the baseline parameters and confounders. *P* values less than 0.05 were considered statistically significant.

## Results

**Fig. 1:**
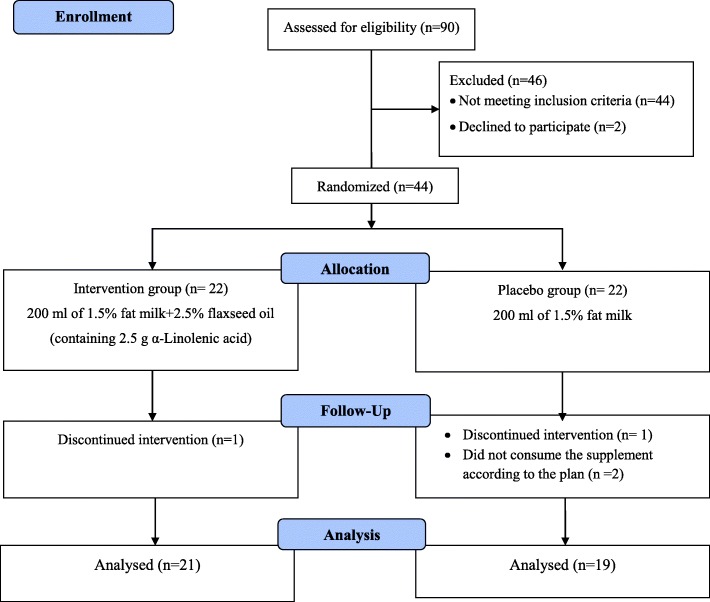
Flowchart of the study

Forty out of 44 patients completed the trial (intervention group, *n* = 21; placebo group, *n* = 19) (Fig. 1). The baseline characteristics of the participants are presented in Table 1. The mean (SD) age of the participants was 55.25 (7.25) years, with 90% of subjects being male. There was no significant difference between the two groups regarding age, sex, weight, BMI, WC, NC, CAD duration, smoking, or physical activity level at baseline (*P* > 0.05). All of the participants consumed aspirin and statin, which led to a non-significant difference between the intervention and placebo groups (*P* > 0.05). Antihypertensive drugs were used by 18 (85.7%) and 15 (78.9%) subjects in the intervention and placebo groups, respectively.

**Table 1:** Baseline characteristics of the study subjects^a^

Variable	Flaxseed oil (*n* = 21)	Placebo (*n* = 19)	*P* value^c^
**Age (years)**	55.67 (6.9)	54.80 (7.8)	0.708
**Sex**^d^**(%)**			
Male	19 (90.5)	17 (89.5)	0.916
Female	2 (9.5)	2 (10.5)	
**Weight (kg)**	86.02 (10.70)	85.66 (10.19)	0.915
**Body mass index (kg/m**^**2**^**)**	30.36 (3.04)	30.70 (3.90)	0.766
**Waist circumference (cm)**	104.42 (7.68)	107.29 (8.63)	0.305
**Neck circumference (cm)**	41.08 (2.53)	41.71 (3.88)	0.576
**CAD duration (years)**	2.6 (1.1)	2.8 (1.7)	0.618
**Medication (%)**			
Statin	21 (100)	19 (100)	1.000^d^
Aspirin	21 (100)	19 (100)	1.000^d^
Anti-hypertensive drugs	18 (85.7)	15 (78.9)	0.689^d^
**SBP (mmHg)**	116.62 (12.13)	112.50 (15.09)	0.352
**DBP (mmHg)**	75.72 (8.51)	79.59 (10.14)	0.216
**Smoking (%)**			
Yes	1 (4.8)	3 (15.8)	0.331^d^
No	20 (95.2)	16 (84.2)	
**Physical activity (METs)**^**b**^	539.74 (45, 2628)	537.95 (99, 1920)	0.991

*CAD* coronary artery diseases, *SBP* systolic blood pressure, *DBP* diastolic blood pressure, *METs* metabolic equivalents

^a^Values are expressed as mean (SD)

^b^Values are expressed as geometric mean (minimum, maximum) and p-value are estimated after log-transformation

^c^Independent samples *t*-test

^d^Values are expressed as frequency (%) and *P* value based on chi-square test

Changes in the fatty acid composition of erythrocyte membrane phospholipids are presented in Table 2. The pre-intervention analysis indicated no significant difference between the two groups in terms of fatty acid content of erythrocyte membrane, including LA, AA, ALA, EPA, and DHA (*P* > 0.05). Within-group analysis after the intervention showed that flaxseed oil supplementation caused a significant reduction in LA (MD = − 4.84, *P* = 0.031) and AA (MD = − 4.03, *P* = 0.026) and an elevation in ALA (MD = 0.37, *P* < 0.001) of membrane phospholipids, compared to the baseline. No significant changes were observed within the groups in terms of EPA and DHA (*P* > 0.05). In the intervention group, the LA content of membrane phospholipids reduced significantly, compared to the placebo group (MD = 2.89, *P* = 0.045, based on ANCOVA adjusted for the baseline values and confounders). Moreover, changes in the ALA content (MD = − 0.35) were significant (*P* < 0.001 based on ANCOVA adjusted for the baseline values and confounders) in the intervention group, compared to the placebo group. However, after 10 weeks of intervention, changes in AA, EPA, and DHA were not significant in the intervention group in comparison with the placebo group (*P* > 0.05).

**Table 2:** Effect of flaxseed oil supplementation on fatty acids status of erythrocytes

Variable	Flaxseed oil (***n*** = 21)	Placebo (***n*** = 19)	MD (95% CI), ***P*** value
**LA (%)**			
Before	14.53 (6.22)	15.59 (8.48)	1.32 (− 4.26 to 6.90), 0.632^a^
After	9.68 (3.25)	12.10 (3.94)	2.89 (0.07 to 5.72), 0.045^b^
MD (95% CI), *P* value^**c**^	− 4.84 (− 9.16 to − 0.53), 0.031	− 3.49 (− 7.18 to 0.19), 0.062	
**AA (%)**			
Before	13.59 (5.64)	11.59 (6.87)	− 1.21 (− 6.07 to 3.65), 0.613^a^
After	9.56 (1.40)	8.75 (1.56)	− 1.06 (− 2.47 to 0.36), 0.135^b^
MD (95% CI), *P* value^**c**^	− 4.03 (− 7.46 to − 0.60), 0.026	− 2.84 (− 6.76 to 1.07), 0.143	
**ALA (%)**			
Before	0.20 (0.12)	0.21 (0.11)	0.01 (− 0.07 to 0.10), 0.679^a^
After	0.56 (0.24)	0.22 (0.10)	− 0.35 (− 0.51 to − 0.20), < 0.001^b^
MD (95% CI), *P* value^c^	0.37 (0.23 to 0.51), < 0.001	0.004 (− 0.08 to 0.09), 0.925	
**EPA (%)**			
Before	1.15 (0.60)	1.08 (0.87)	− 0.28 (− 0.84 to 0.27), 0.301^a^
After	1.06 (0.72)	0.87 (0.50)	− 0.29 (− 0.96 to 0.37), 0.364^b^
MD (95% CI), *P* value^c^	− 0.09 (− 0.51 to 0.32), 0.612	− 0.21 (− 0.65 to 0.22), 0.305	
**DHA (%)**			
Before	1.43 (0.87)	1.24 (0.76)	− 0.20 (− 0.83 to 0.43), 0.520^a^
After	1.36 (0.44)	1.29 (0.46)	− 0.08 (− 0.52 to 0.36), 0.704^b^
MD (95% CI), *P* value^c^	− 0.07 (− 0.80 to 0.65), 0.822	0.05 (− 0.35 to 0.45), 0.796	

Values are expressed as mean (SD)

*LA* linoleic acid, *AA* arachidonic acid, *ALA* α-linolenic acid, *EPA* eicosapentaenoic acid, *DHA* docosahexaenoic acid, *MD* mean difference

^a^Independent samples *t*-test

^b^Adjusted for baseline values, weight differences, and energy and fat intake changes using the analysis of covariance (ANCOVA) test

^c^Paired-samples *t*-test

Table 3 presents the serum AEA levels before and after the intervention. At baseline, no significant difference was observed between the two groups (*P* > 0.05). After 10 weeks of intervention, the AEA levels in the intervention group showed a marked decline, compared with the placebo group following adjustment for confounding factors (MD = 0.64, *P* = 0.016). The within-group analysis indicated that changes in AEA levels were significant in the intervention group (MD = − 0.71, *P* = 0.001), but not the placebo group (*P* > 0.05).

**Table 3:** Effect of flaxseed oil supplementation on AEA status

Variable	Flaxseed oil (***n*** = 21)	Placebo (***n*** = 19)	MD (95% CI), ***P*** value
**AEA (ng/ml)**			
Before	5.45 (2.98)	6.76 (2.72)	1.31 (− 0.54 to 3.17), 0.158^a^
After	4.73 (2.67)	6.44 (2.44)	0.64 (0.12 to 1.15), 0.016^b^
MD (95% CI), *P* value^**c**^	− 0.71 (− 1.08 to − 0.34), 0.001	− 0.32 (− 0.70 to 0.05), 0.091	

Values are expressed as mean (SD)

*AEA* N-arachidonoyl ethanolamine, *MD* mean difference

^a^Independent samples *t*-test

^b^Adjusted for baseline values, weight differences, energy intake changes and fat intake changes using the analysis of covariance (ANCOVA) test

^c^Paired-samples *t*-test

**Fig. 2:**
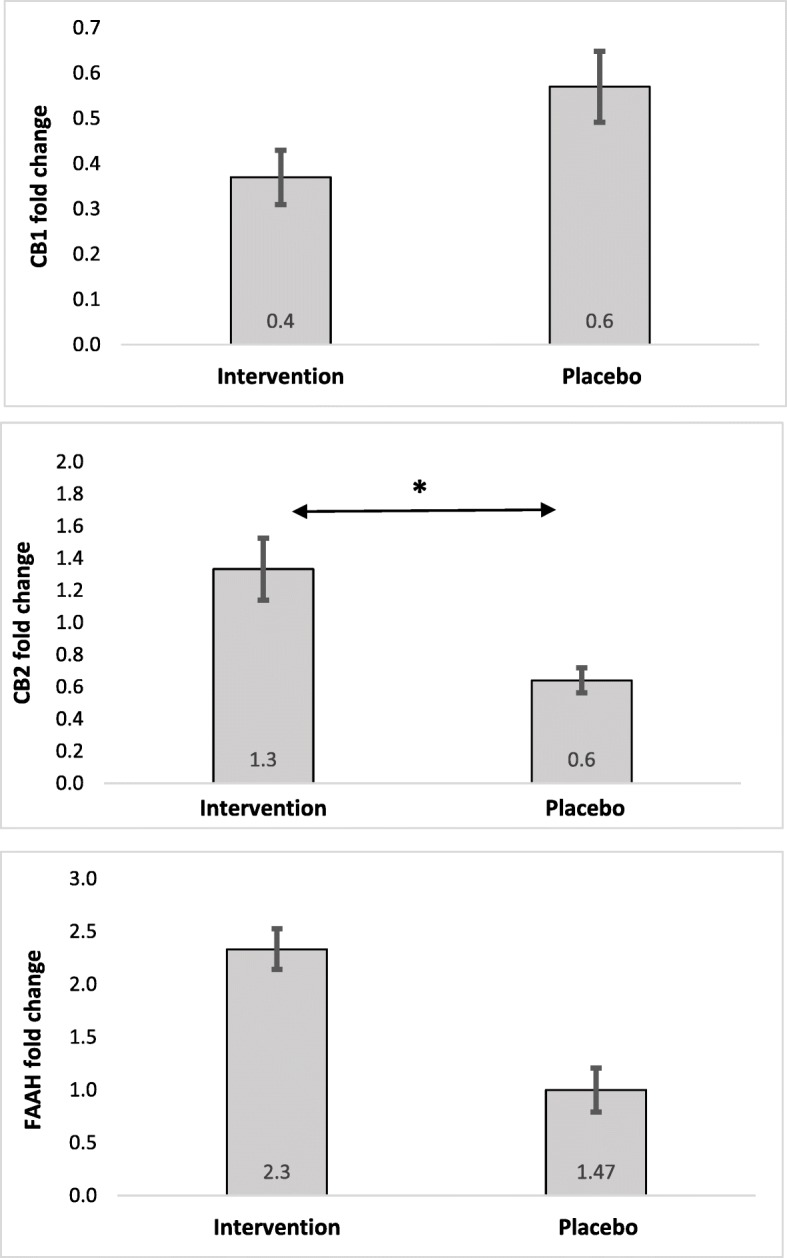
CB1, CB2, and FAAH fold change in the study groups. Each point represents mean (SD). Data analysis was performed using independent sample *t*-test (*P < 0.05). CB1: cannabinoid receptor type-1; CB2: cannabinoid receptor type-2; FAAH: fatty acid amide hydrolase

As presented in Fig. 2, a non-significant reduction was found in CB1 fold changes (fold change 0.40, *P* = 0.059) following supplementation with flaxseed oil, compared with the placebo group. However, dietary supplementation with flaxseed oil significantly increased the CB2 fold changes, compared with the placebo group (fold change 1.30, *P* = 0.003). On the other hand, changes in FAAH mRNA expression were not significant (fold change 2.30, *P* = 0.074).

## Discussion

A growing number of studies have indicated that consumption of flaxseed oil and its omega-3 fatty acid content exerts cardiovascular protective effects [1, 31–33]. However, the exact mechanism through which these fatty acids exert their beneficial effects on cardiac health is unknown. ECS functions within cardiovascular tissues [27], and ECS dysregulation has been reported in both obese and CAD individuals, with specific clinical effects on systemic lipid metabolism, restenosis, and inflammation [34–36]. CB1 antagonists are potential targets for ameliorating clinical conditions related to cardiometabolic disease [37–39]. In this study, we tried to determine if the favorable effects of flaxseed oil can be attributed to its role in the modulation of endocannabinoids in CAD patients, possibly by limiting the availability of biosynthetic precursors for endocannabinoids (i.e., LA and AA) and increasing omega-3 fatty acid content of membrane phospholipids.

In the present study, 5 g of flaxseed oil supplementation per day, rich in ALA, led to the significant reduction of LA and significant elevation of ALA in erythrocyte phospholipids. The EPA and DHA contents of membrane phospholipids were not significantly different, compared with the baseline. Conversion of ALA to LC-omega-3 fatty acids has been determined in several clinical trials [13, 15, 16]. It should be noted that the supplemented dose of flaxseed oil in the present study was lower than other trials. However, Barceló-Coblijn reported that 2.4 g/day or 3.6 g/day of flaxseed oil was sufficient to increase the omega-3 fatty acid content of erythrocyte membrane phospholipids [9]. Apparently, similar or greater changes in various tissues depend on the need of these tissues for LC-fatty acids [14]. In the present study, the ALA to LA ratio increased following flaxseed oil supplementation. However, the absolute ALA content was not very high which might be the main reason for the insignificant bioconversion to longer homologs rather than the ALA to LA ratio [40].

Our findings indicated that flaxseed oil supplementation led to a marked reduction in the serum AEA levels. The literature review revealed that researchers have focused on the effects of animal-derived oils containing omega-3 fatty acid on the endocannabinoid synthesis, endocannabinoid signaling, and its health consequences. In various animal studies [6, 41–47] and two human studies [48, 49], dietary exposure to EPA and DHA (e.g., fish oil, krill oil, and pure EPA and DHA) induced a significant reduction in AEA and 2-AG in plasma and different tissues. In this regard, a study by Demizieux et al. showed that incorporation of flaxseed oil, as a plant source of omega-3 fatty acids, to the diet of mice led to a marked reduction in the liver endocannabinoid level [33].

In contrast to the abovementioned studies, Piscitelli F. et al. [50] revealed that krill oil not only failed to decrease hepatic AEA and 2-AG levels, but also increased the endocannabinoid tone. This discrepancy can be explained by different fatty acid content of high-fat diet (HFD) [51]. The HFD used in the study by Piscitelli F. et al. contained very low levels of LA, which led to a low AA content in hepatic phospholipids, as precursor of endocannabinoid biosynthesis. The findings of previous studies, indicating the higher level of LA in HFD, confirm this explanation [52–55]. In the current study, significant reduction of LA and elevation of ALA could be responsible for the lower AEA level in the intervention group. However, Demizieux believes that competition between omega-3 and omega-6 for elongation and desaturation in endocannabinoid biosynthesis might be more important than a net supply of omega-6 as an endocannabinoid biosynthesis precursor [33].

In the present trial, flaxseed oil exerted the greatest effects on CB2 receptor expression; this effect might be related to the high content of ALA. A marginally significant reduction was observed in the mRNA expression of CB1 (*p* = 0.059). Previous studies have examined dietary omega-3 fatty acids in ECS modulation in patients with metabolic disorders. Kim et al. demonstrated that DHA treatment of C57BL/6 J mice increased the mRNA level of cannabinoid receptors and metabolizing enzymes in the muscles and fat mass of mice, suggesting a compensatory response to increased DHEA levels with lower affinity to CB1 and CB2. Also, reduced activity of ECS was associated with the increased mRNA expression of adiponectin, resulting in the lower fat mass and favorable metabolic consequences in the DHA-fed group [46].

Recently, Demizieux suggested that early incorporation of dietary omega-3 in the form of flaxseed oil led to decreased mRNA expression of CB1 and lipogenic and gluconeogenic enzymes; this finding indicates the improved insulin resistance and glycemic control. The increased CB2 mRNA expression and decreased mRNA expression of lipoprotein lipase and fatty acid synthase suggest the anti-inflammatory and anti-proliferative activities of flaxseed oil in the adipose tissues of transgenic mice [33]. It is obvious that the positive effects of dietary treatment may depend on the nature and source of fatty acids in the diet [56]. Flaxseed oil has been reported to exert its anti-inflammatory effects by lowering the level of high-sensitivity C-reactive protein [11], interleukin-1 and TNF-α gene expression [10]. In line with the study by Demizieux et al. [33], the present study confirmed this effect due to increased CB2 gene expression and reduced CB1 gene expression.

Our findings related to FAAH indicated a marginally significant upregulation (*P* = 0.074), suggestive of the regulation of endocannabinoid degradation pathway in favor of reduction in AEA. Our results are consistent with previously published in vivo data, indicating the upregulation of FAAH and downregulation of CB1 following flaxseed oil treatment [33]. The FAAH mRNA expression increased markedly in fat-1 mice, the characteristic of which is great ability to biosynthesize omega-3 from omega-6. Therefore, it can be concluded that dietary history contributes to ECS regulation relative to the fatty acid content of diet. The link between ECS and de novo lipogenesis can also lead to this conclusion. Liu et al. [57] proposed that stearoyl coA desaturase 1 (SCD1), which generates monounsaturated fatty acids (MUFAs), is an inhibitor of liver FAAH, which leads to elevation of AEA in the liver. Based on the study by Demizieux [33], flaxseed oil supplementation caused a decrease in hepatic MUFA levels, which suggests changes in SCD1 function, its downregulation, and increased FAAH activity.

However, our study is in contrast to a study by Batetta [58], which showed that FAAH activity was not significantly different between intervention (fish oil or krill oil) and placebo groups. It was previously shown that FAAH expression and activity were associated with the AEA content in the heart, liver, and adipose tissue of obese people. The reduced level of AEA in the current study may be partly attributed to FAAH overexpression.

## Conclusions

The present results showed that flaxseed oil in milk emulsion (containing 2.5 g of ALA) could decrease AEA levels and increase CB2 mRNA expression. Our findings support the growing belief that flaxseed oil is a functional food with cardioprotective properties by reducing the ECS overactivity.

## Data Availability

The dataset used and analyzed during the current study is available from the corresponding author on reasonable request.
